# Invasive Aspergillus fumigatus Sinusitis as a Mucormycosis Mimic in a Liver Transplant Recipient

**DOI:** 10.7759/cureus.115463

**Published:** 2026-08-30

**Authors:** Sloane Parker, Arta Kasaeian, Sahar Samimi, Andrew Caruso

**Affiliations:** 1 Department of Internal Medicine, Baylor College of Medicine, Houston, USA; 2 Department of Radiology, Baylor College of Medicine, Houston, USA

**Keywords:** aspergillus fumigatus, immunocompromised host, invasive fungal sinusitis, liver transplant, mucormycosis, mucormycosis mimic, transplant recipient

## Abstract

Invasive fungal sinusitis is a serious infection in immunocompromised patients, and distinguishing *Aspergillus *from *Mucorales* is diagnostically challenging but clinically critical, as the two organisms require markedly different treatment. A 56-year-old man with alcohol-related cirrhosis status post orthotopic liver transplantation, maintained on tacrolimus and prednisone with well-controlled type 2 diabetes, presented with progressive left facial pain, numbness, and swelling. Despite evaluation by otolaryngology, dental extraction, and antibiotic therapy, his symptoms worsened. Initial imaging was non-diagnostic, and invasive fungal sinusitis was suspected, with mucormycosis considered most likely given his immunosuppression and diabetes. However, surgical biopsy and culture ultimately revealed *Aspergillus fumigatus*. He underwent surgical debridement and was treated with liposomal amphotericin B followed by oral posaconazole, with improvement in swelling, though residual facial numbness and dental pain persisted. This case illustrates the diagnostic challenge of invasive fungal sinusitis in transplant recipients, in whom *Aspergillus* can closely mimic mucormycosis on imaging and frozen section. Clinicians should maintain a broad differential for facial pain in immunocompromised patients to optimize outcomes.

## Introduction

Invasive fungal sinusitis is an uncommon but serious infection that occurs predominantly in immunocompromised patients. It is characterized by fungal invasion of the sinonasal mucosa with potential extension into adjacent structures, such as the bone, orbit, and brain [1]. Among the causative organisms, *Mucorales* and *Aspergillus* species are the most frequently implicated, along with *Fusarium*, *Scedosporium*, and dematiaceous molds [2-4]. Distinguishing between these organisms is clinically important, as optimal antifungal therapy differs significantly. While galactomannan and polymerase chain reaction (PCR) assays are useful in pulmonary fungal disease, head and neck presentations often rely on histopathology and culture, which may delay definitive diagnosis.

Frozen section is frequently used intraoperatively to confirm the presence of fungal invasion and to guide the extent of debridement, but it has important limitations for organism identification. Freezing artifact can distort hyphal architecture, and the morphologic features that distinguish *Mucorales* from *Aspergillus* may be difficult to appreciate on a rapid intraoperative preparation. Permanent histopathology allows more detailed assessment of hyphal morphology and of tissue and vascular invasion, while culture remains necessary for definitive species-level identification. Discordance between the initial frozen-section impression and final culture is therefore not uncommon and can have direct therapeutic consequences.

We present the case of a liver transplant recipient with chronic invasive fungal sinusitis initially presumed to be mucormycosis on frozen section, but ultimately confirmed to be *Aspergillus fumigatus* on culture. This case highlights the diagnostic challenges and management considerations in post-transplant fungal sinusitis.

## Case presentation

A 56-year-old man who had undergone orthotopic liver transplantation for alcohol-related cirrhosis, maintained on tacrolimus and prednisone and with well-controlled type 2 diabetes, presented with progressive left facial pain, numbness, and swelling following a dental extraction. He completed an outpatient course of amoxicillin-clavulanate without improvement. Ear, nose, and throat (ENT) evaluation, including nasal endoscopy and CT imaging a month later, was unremarkable. His symptoms progressed over the following weeks, and by early next month, he developed numbness in the right V2 distribution, periorbital swelling, and ocular drainage and was admitted to our hospital.

Malignancy was considered early in the course given the progressive nature of symptoms, bone erosion, and infiltrative imaging findings that can mimic sinonasal carcinoma or lymphoma. Neuropathic pain was also initially favored by the initial outpatient providers given the lack of abnormalities on early imaging and endoscopy and the patient's facial pain distribution. Once he was admitted with periorbital swelling and ocular drainage, invasive fungal sinusitis was suspected, and mucormycosis was considered most likely given his immunosuppression from liver transplantation and underlying diabetes.

During endoscopic sinus surgery on the second day of hospitalization in the same month, fungal-appearing tissue was identified directly overlying the posterior maxillary wall and was removed and sent for tissue culture. The underlying bone was grossly necrotic and was resected and sent for permanent pathology, and inflamed mucosa was submitted for intraoperative frozen section, which confirmed fungal hyphae with morphology suspicious for a *Mucorales* infection. Necrotic muscle and involvement of the infraorbital nerve were also encountered on exploration.

Repeat CT and MRI (Figure 1) demonstrated bony erosion of the posterior maxillary wall, extrasinus soft-tissue extension into the pterygopalatine fossa, and orbital involvement.

**Figure 1:**
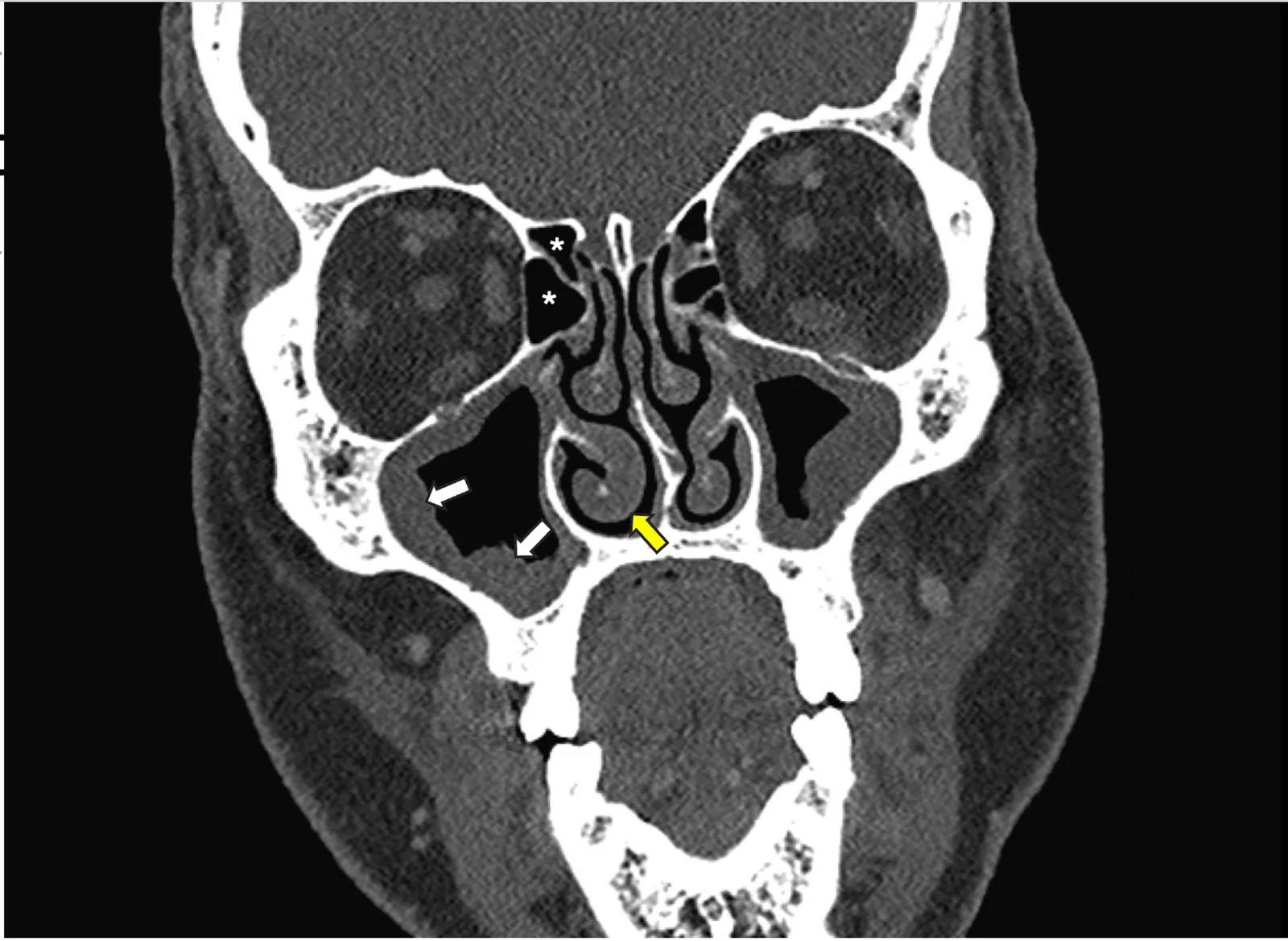
Coronal contrast-enhanced maxillofacial computed tomography (CT) Demonstrates diffuse mucosal thickening within the right maxillary sinus (white arrows) and right inferior nasal meatus (yellow arrow), with osseous erosion of the right ethmoidal air cells (white stars).

Together with the intraoperative findings of necrotic bone, muscle, and nerve, and the demonstration of fungal hyphae within tissue on frozen section, these features supported a diagnosis of invasive fungal sinusitis. The permanent surgical pathology report was not available for review; consequently, the specific histopathologic features underlying the intraoperative *Mucorales* impression could not be further characterized, and the presence or absence of vascular invasion could not be determined. Laboratory studies showed stable graft function and therapeutic tacrolimus levels.

Given the patient's immunosuppression from liver transplantation and diabetes, together with the necrotic sinonasal tissue and the intraoperative report describing a frozen-section impression of *Mucorales*, mucormycosis was initially strongly suspected. Surgical cultures obtained from three sinus sites (left pterygopalatine fossa, left maxillary sinus, and left sphenoid sinus) ultimately all grew *Aspergillus fumigatus*, establishing invasive aspergillosis rather than mucormycosis as the etiology despite the initial intraoperative frozen-section impression. This discordance underscores the limited reliability of frozen-section morphology for distinguishing *Mucorales* from *Aspergillus*, and the importance of culture for definitive speciation.

The patient was started empirically on intravenous liposomal amphotericin B with topical amphotericin B sinus irrigations, given the initial intraoperative concern for mucormycosis, along with broad-spectrum antibacterial coverage with vancomycin and meropenem. Antibacterial agents were discontinued once surgical cultures confirmed *Aspergillus fumigatus*. Antifungal therapy was transitioned to intravenous posaconazole and subsequently to oral posaconazole 300 mg daily due to drug shortage, with therapeutic drug monitoring targeting a trough concentration greater than 1-1.5 mg/L. Hepatology reduced immunosupression, including his prednisone dose to 10 mg daily and tacrolimus to 2 mg twice daily. Troughs and liver function tests were collected daily to monitor interaction between tacrolimus and posaconazole. 

Surgical management included right maxillary antrostomy, total ethmoidectomy, sphenoidotomy, and exploration of the pterygopalatine fossa with removal of necrotic tissue. Supportive care focused on tight glucose control, correction of electrolyte abnormalities, and ongoing monitoring of transplant function.

Outcome and follow-up

Following treatment, the patient's periorbital swelling improved substantially, and he was discharged on day 13 of hospitalization with long-term oral posaconazole 300 mg daily and amphotericin rinses three times daily. He continued reduced immunosupression dosing and tacrolimus trough levels collected weekly for the first three months post-hospitalization. At that time, CT showed stable thickening of the polypoid mucosa and the pterygomaxillary fissure into the infratemporal fossa/masticator space, as well as along the right infraorbital nerve foramen consistent with stable disease. Amphotericin rinses and posaconazole continued with monthly collection of posaconazole and tacrolimus trough levels. At six months post-hospitalization, a repeat MRI showed improvement of the sinuses with decreased enhacement and resolved mucosal opacification. At that time he continued to have residual symptoms included persistent numbness in the right V2 distribution and localized dental pain, though graft function remained stable with no recurrent fevers or systemic signs of infection. The patient completed one year of the antimicrobials with final CT showing resolution of disease.

## Discussion

Invasive fungal sinusitis remains a life-threatening complication in immunocompromised patients, particularly solid-organ transplant recipients. Invasive fungal sinusitis due to *Aspergillus* species is increasingly recognized in the literature. In organ transplant recipients specifically, *Aspergillus* is consistently reported as the predominant cause of invasive fungal infection, with mucormycosis representing a smaller but clinically significant proportion of cases [1,2,5]. In multicenter studies of transplant recipients with invasive mycelial fungal infections, *Aspergillus* accounted for approximately 70% of cases, while *Mucorales* species were responsible for fewer than 6% [6]. Mucormycosis accounts for only 2-8% of invasive fungal infections among recipients of solid-organ and allogeneic stem cell transplants. In contrast, the Infectious Diseases Society of America notes that invasive *Aspergillus* infection occurs in up to 19% of solid organ transplant recipients, with incidence varying by organ type [2,7].

The radiologic presentation of invasive *Aspergillus* sinusitis can closely resemble that of mucormycosis, with both entities showing bony erosion, periantral fat involvement, and extrasinus extension on imaging [8]. These findings are not pathognomonic and may also resemble sinonasal malignancy, necessitating histopathologic confirmation for a definitive diagnosis [9]. Some clinical features can help distinguish *Aspergillus* from mucormycosis, including a greater tendency for mucormycosis to invade the orbit bilaterally and more aggressively; however, these are trends rather than reliable discriminators [10]. This case illustrates the diagnostic pitfalls that can arise in invasive fungal sinusitis: reliance on frozen section, imaging, or clinical presentation alone can misdirect therapy, underscoring the need for culture and histopathologic confirmation before finalizing a treatment strategy.

Correctly distinguishing mucormycosis from aspergillosis has important therapeutic implications. Mucormycosis is classic, rapidly progressive, and angioinvasive, requiring urgent surgical debridement and high-dose liposomal amphotericin B, whereas *Aspergillus* infections often follow a more indolent, chronic course in transplant recipients and respond well to triazole therapy such as posaconazole or voriconazole [1,11]. In this case, posaconazole was chosen because of better tolerability and reduced associated risk of hepatobiliary injury [12]. Optimal management requires synergy between surgical and medical approaches, combining prompt, aggressive debridement with targeted antifungal therapy.

## Conclusions

Chronic invasive fungal sinusitis is an uncommon but important diagnostic consideration in immunocompromised patients presenting with persistent facial pain or neuropathic symptoms, even when initial imaging or endoscopic evaluation is unrevealing. Its indolent presentation can closely mimic malignancy or primary neuropathic disorders, contributing to delays in diagnosis. This case also highlights the limitations of relying on frozen section analysis alone, as definitive fungal culture and permanent histopathology remain essential for accurate diagnosis and timely initiation of appropriate antifungal therapy.
